# Egg Production and Bone Stability of Local Chicken Breeds and Their Crosses Fed with Faba Beans

**DOI:** 10.3390/ani10091480

**Published:** 2020-08-22

**Authors:** Tanja Nolte, Simon Jansen, Ingrid Halle, Armin Manfred Scholz, Henner Simianer, Ahmad Reza Sharifi, Steffen Weigend

**Affiliations:** 1 Department of Animal Sciences, Animal Breeding and Genetics Group, University of Goettingen, 37075 Goettingen, Germany; hsimian@gwdg.de (H.S.); rsharif@gwdg.de (A.R.S.); 2 Center for Integrated Breeding Research, University of Goettingen, 37075 Goettingen, Germany; steffen.weigend@fli.de; 3 Institute of Farm Animal Genetics, Friedrich-Loeffler-Institut, 31535 Neustadt, Germany; 4 Institute of Animal Nutrition, Friedrich-Loeffler-Institut, 38116 Braunschweig, Germany; ingrid.halle@fli.de; 5 Livestock Center of the Faculty of Veterinary Medicine, Ludwig-Maximilians-University Munich, 85764 Oberschleissheim, Germany; armin.scholz@lvg.vetmed.uni-muenchen.de

**Keywords:** bone stability, crossbreeding, egg weight, faba bean, laying performance, local breeds, vicin

## Abstract

**Simple Summary:**

Poultry production systems are currently facing important issues like animal welfare, the environmental impact of soy imports from overseas and the decline in genetic diversity. The current study aims at testing an alternative production system that could provide niche markets with regional poultry products. Six different chicken genotypes were tested in this study regarding egg production traits and bone stability. As a regional alternative to soy, two varieties of locally grown faba beans have been used in the animals’ diets. A limited adverse effect of the vicin-rich faba bean diet on egg weight was observed. The crossbred chicken of the local breed Bresse Gauloise with the commercial laying hen White Rock seems to be the most promising.

**Abstract:**

Poultry production is raising concerns within the public regarding the practice of culling day-old chicks and the importation of soy from overseas for feedstuff. Therefore, an alternative approach to poultry production was tested. In two consecutive experiments, two traditional chicken breeds, Vorwerkhuhn and Bresse Gauloise, and White Rock as a commercial layer genotype as well as crossbreds thereof were fed diets containing either 20% vicin-rich or vicin-poor faba beans, though addressing both subjects of debate. Hen performance traits and bone stability were recorded. All parameters were considerably influenced by the genotype with White Rock showing the significantly highest (*p* < 0.05) laying performance (99.4% peak production) and mean egg weights (56.6 g) of the purebreds, but the lowest bone breaking strength (tibiotarsus 197.2 N, humerus 230.2 N). Regarding crossbreds, the Bresse Gauloise × White Rock cross performed best (peak production 98.1%, mean egg weight 58.0 g). However, only limited dietary effects were found as only the feeding of 20% vicin-rich faba beans led to a significant reduction of egg weights of at most 1.1 g (*p* < 0.05) and to a significant reduction of the shell stability in the crossbred genotypes. In terms of dual-purpose usage, crossing of Bresse Gauloise with White Rock seems to be the most promising variant studied here.

## 1. Introduction

Public demands placed on agriculture have changed drastically in recent years. While only a few decades ago, the main goal was to produce sufficient good quality food at a favorable price, today’s farmers are encouraged to consider more ethical issues, such as animal welfare and the conservation of natural and climatic resources.

One major ethical concern considers the killing of day-old male chicks of the layer lines due to economic reasons as very critical leading to a ban (of this procedure) in Germany as soon as alternative solutions are ready for practical use [1]. One possible alternative could be the use of dual-purpose chickens, where the hens can be used for egg production while the cocks are fattened for meat production. The main problem of this production system is the negative genetic correlation between laying and fattening performance [2,3], making it difficult to improve both traits in the same line. However, crossbreeding could improve the chickens’ overall performance, as shown in local chicken breeds by Götze and von Lengerken [4]. Moreover, the use of local chicken breeds in agricultural production contributes to the conservation of these breeds as genetic resources [5].

Animal welfare is an issue that is becoming increasingly important in poultry production. One major problem the egg industry facing currently is the high incidence of laying hens with skeletal disorders [6,7], both in intensive and extensive housing systems. It has been shown that not only nutrition and husbandry of the hens, but also genetics play an important role in bone stability [8]. While the majority of studies have investigated bone stability in contemporary laying hybrids, the number of such studies conducted in local chicken breeds is very limited. However, it was found that bone characteristics differ considerably between genotypes [9], which is why findings from high performing lines can probably not fully be transferred to local breeds. So far, there has been little discussion about how purebred local chicken breeds and their crossbreds differ in terms of bone characteristics.

In order to provide the meat producing sector with high-quality protein feed, the EU imports huge amounts of soybeans from overseas, namely 15.1 million metric tons in the season 2019/20 [10]. While the use of genetically modified seeds is common in the main producing countries USA and Brazil, this is seen critically by European consumers [11]. In addition, the negative environmental impact of the soybean production especially in South America [12] leads to a reduction of soybean imports in favor of regional protein crops in Germany [13]. A suitable regional protein plant is the faba bean (*Vicia faba* L.) [14]. However, until now, anti-nutritive substances, like for example, the endogenous glycosides vicin and convicin (together abbreviated as VC) limit the use of faba beans in animal nutrition. Today few VC-poor varieties are available, for example, the variety Tiffany, but the majority of cultivated faba beans is still VC-rich, as less than one-third of the cultivation area used for seed production is planted with VC-poor varieties [15].

The influence of faba bean feeding and of VC on the performance and health of laying hens has been studied for decades. However, the results of the studies and the recommended maximum levels for hen diets vary greatly and are usually evaluated with commercial chicken genotypes. Jeroch et al. [16] recommend maximum levels of 10% for conventional faba bean varieties and 20% of varieties with reduced VC content during the laying period. Described consequences of higher faba bean fractions in the feed are, for example, increased animal mortality [17], reduction of laying performance [17,18], and of egg weight [19,20,21]. In contrast, Daenner [22] did not find any performance reductions feeding a diet with 30% vicin-rich faba beans.

Addressing alternative ways of poultry production for niche markets, the present study aims at evaluating different egg production and bone stability traits of two local chicken breeds, one commercial line, and of their crossbreds, while feeding regional faba beans with differing VC-contents. By comparing pure and crossbreds, we aim to investigate whether the high performing line will increase the performance of the local breeds to such an extent that it will become economically viable for poultry production.

## 2. Materials and Methods

### 2.1. Ethical Note

The current experiments were performed in accordance with the German Animal Welfare Law and approved by the Lower Saxony State Office for Consumer Protection and Food Safety (LAVES) (33.19-42502-04-17/2600).

### 2.2. Stock and Husbandry

The study included three purebred and three crossbred genotypes of domestic chicken (*Gallus gallus domesticus*). The purebred genotypes were Vorwerkhuhn (VH), Bresse Gauloise (BG) and White Rock (WR). VH is a local chicken breed from Germany, which was originally bred for dual-purpose usage. The BG hens originate from the Bresse region in the south of France Burgundy County, where they are marketed as a delicacy with protected designation of origin (PDO) [23]. They achieve an annual laying performance of around 250 eggs [24]. WR is a commercial layer line from Lohmann Tierzucht GmbH (Cuxhaven, Germany), which originates, amongst others, from Plymouth Rock chicken. As high performing line, WR is a founder population of some modern laying hybrids with brown eggshell color. To build up grandparent stocks, birds of the VH and BG breed were provided by fancy breeders of a conservation flock for poultry species. Based on these animals, parent stocks of VH and BG purebreds were generated, which were then used to generate the test animals. The WR hens were provided as day-old chicks by Lohmann Tierzucht GmbH (Cuxhaven, Germany).

Two consecutive experiments were conducted. In experiment A, purebred hens of the VH, BG and WR breeds were tested. Experiment B dealt with crossbreds thereof, which were generated by crossing cocks of VH and BG either with hens of BG or WR, resulting in three crossbreds: VH × BG (VBG), VH × WR (VWR) and BG × WR (BWR).

Hatching and rearing procedures were identical in both experiments. After being wing-tagged for identification, blood samples were taken from VH and BG chicks within the first week of life for sex determination via DNA analysis [25]. Female WR chicks were provided by the breeding company. In the case of experiment B, blood sampling and molecular sexing was only done for BWR chicks, because VWR and VBG chicks could be sexed visually based on different plumage color. Commercially available complete feeding stuffs for chicks (until 6 weeks of age; 11.4 MJ AMEn/kg DM, 180.0 g/kg crude protein, 26.1 g/kg crude fat, 37.5 g/kg crude fiber, 56.0 g/kg crude ash, 7.8 g/kg calcium, 4.7 g/kg phosphorous) and pullets (from 7 to 17 weeks of age; 11.0 MJ AMEn/kg DM, 145.0 g/kg crude protein, 37.0 g/kg crude fat, 65.05 g/kg crude fiber, 59.0 g/kg crude ash, 10.0 g/kg calcium, 6.0 g/kg phosphorous), as well as water were offered ad libitum. A standard lighting program was applied to the birds, where day length increased gradually from 8 h (8th weeks of age) to 14 h (23rd week of age). After hatch at the Institute of Animal Welfare and Animal Husbandry of the Friedrich-Loeffler-Institut (Celle, Germany), the chicks were raised in a floor housing system. At 7 weeks of age, all birds of the respective experiment were transferred to floor pens at the Institute of Farm Animal Genetics of the Friedrich-Loeffler-Institut (Mariensee, Germany), which were later used as experimental sites. The pens of 12.5 m^2^ were equipped with wood chips, feeding and drinking troughs, a wooden perch, a dust bath, and nine laying nests.

### 2.3. Experimental Procedure

**Figure 1:**
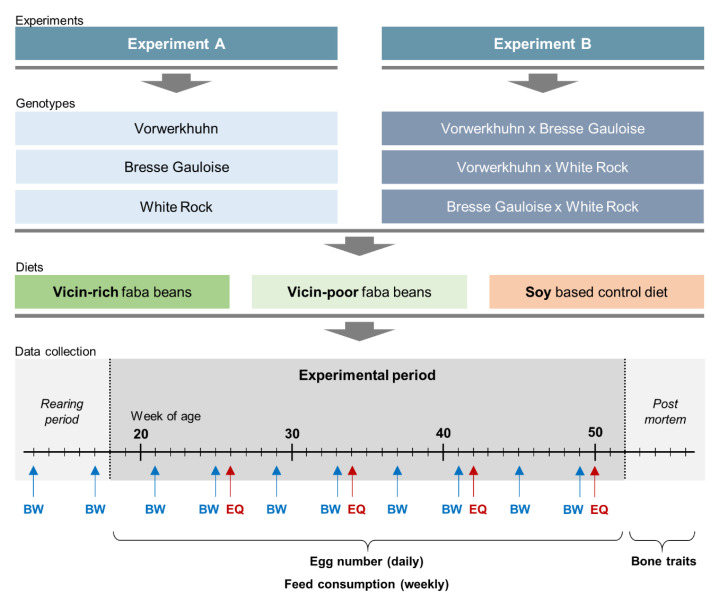
Schematic illustration of the experimental setup. In experiment A, three purebred chicken genotypes were allocated to one of three diets containing either faba beans differing in their vicin content or soybean. Experiment B comprised three crossbred genotypes, which were allocated to the same diets. In experiment A (B), 120 (132) hens per genotype were tested with two replicates per genotype × diet combination consisting of 20 (22) hens each. The data collection was identical in both experiments. Data on egg number, egg quality (EQ), feed consumption and body weight (BW) were collected as indicated. Post mortem, bone morphometry, bone mineral density, bone breaking strength and the cortical bone proportion were assessed.

The experimental setup is shown in Figure 1. In both experiments, the testing period lasted from 18th until 52nd week of age. The hens were subjected to three different feeding treatments. While two diets contained faba beans as an alternative source of protein, the third diet was a soybean-based standard feed as control (Soy). In order to examine the effect of anti-nutritive substances on performance and bone characteristics, the experimental diets contained either 20% of the VC-rich faba bean variety Fuego (VC+) or 20% of the VC-poor variety Tiffany (VC−). To meet the nutritional requirements of the hens without soybean meal, 21% blue sweet lupines (*Lupinus angustifolius* cv. Boruta) were added to all diets. The protein plants were produced GMO-free in Germany. The composition of the diets is specified in Table 1. The changeover to the layer diets has been progressively implemented during the 17th week of life. From the beginning of the 18th week, all hens were fed exclusively with the respective layer diet. A total number of 756 hens entered both experiments. In experiment A, 120 purebred hens per genotype, i.e., a total of 360 hens, were allocated to 18 floor pens (2 × 9) of 20 hens each, whereas in experiment B there were 132 crossbred hens per genotype, resulting in 22 hens per pen and 396 in total. Given the three genotypes per experiment and the three different diets, nine groups of genotype × diet combinations were formed, resulting in 40 purebred or 44 crossbred hens for each experimental group (genotype × diet combination). The housing conditions were the same as described above for the rearing period.

Data collection included performance traits and bone characteristics. Egg production was recorded daily at pen-level, with each observation representing the egg production of 40 (purebreds) or 44 hens (crossbreds). Laying performance was calculated by dividing the total number of eggs by the number of hens present at that day. Feed consumption (g) was recorded weekly at pen-level by weighing the remaining feedstuff. Starting at the 13th week of life, individual body weights (g) of the hens were recorded every four weeks using a digital table scale with a weighing accuracy of 0.1 g (CPA 16001S, Sartorius, Göttingen, Germany). Egg quality was analyzed at four points in time (week 26, 34, 42 and 50). At each point in time, 96 randomly selected eggs from four consecutive days were analyzed per genotype x diet combination. Egg weight (g) was recorded using a digital table scale (CPA 16001S, Sartorius, Goettingen, Germany). Eggshell breaking strength (N) was determined using a texture analyzer (TA.XTplus, Stable Micro Systems, Hamilton, MA, USA) equipped with a 50 N (Newton) load cell showing the maximum load in N that was required to break the eggshell. Eggshell weight (g) was determined after emptying the egg with a spoon and drying the shell for 30 s in a microwave (800 watt). After removing the shell membranes, equatorial eggshell thickness (mm) was measured using a caliper with an accuracy of 0.01 mm.

All hens were sacrificed by carbon dioxide inhalation during the 52nd week of age. The tibiotarsi of both sides and the left humerus were dissected and relieved from muscles and tendons. Bone weight (g), length (mm) and thickness (mm) were recorded and the bones were vacuum-packed and stored frozen (−20 °C) until further examination. Bone mineral density was examined by dual energy X-ray absorptiometry (GE Lunar *i*DXA scanner, GE Healthcare, Solingen, Germany) as described by Jansen et al. [9]. Bone breaking strength (N) of left tibiotarsus and humerus were assessed at the mid-diaphyseal region via three-point bending test (Instron Materials Testing System, Instron Corporation, Canton, MA, USA) using a 5 kN load cell. The span length was 40 mm (humerus) or 80 mm (tibiotarsus). The right tibiotarsus was used to measure diaphyseal cortical bone proportion (%) planimetrically [26].

### 2.4. Statistical Analyses

The data were analyzed separately for experiments A and B using the statistical program SAS (SAS 9.3, SAS Institute Inc., Cary, NC, USA). The separate analysis was chosen, because the two experiments were performed in consecutive years, and an overlap between year- and genotype-effect was not safe to exclude. Therefore, it was also not possible to distinguish between heterosis and year effect.

For the analysis of hen body weight and laying performance, a polynomial growth function was applied according to the model

(1) $$Y_{ijkl}=\mu +G_{i}+D_{j}+G_{i}D_{j}+\overset{5}{\underset{v=1}{\sum }}b_{rv}{\left(A_{ij}\right)}^{v}+\overset{2}{\underset{v=1}{\sum }}b_{sv}G_{i}{\left(A_{ij}\right)}^{v}+\overset{2}{\underset{v=1}{\sum }}b_{tv}D_{j}{\left(A_{ij}\right)}^{v}\;+\overset{2}{\underset{v=1}{\sum }}b_{uv}G_{i}D_{j}{\left(A_{ij}\right)}^{v}+p_{k}+e_{ijkl}$$

where $Y_{ijkl}$ is the body weight respectively of laying performance, μ is the overall mean, $G_{i}$ is the fixed effect of the genotype (i = 1 to 3), $D_{j}$ is the fixed effect of the diet (j = 1 to 3), $G_{i}D_{j}$ is the interaction of genotype x diet, $b_{rv}$ are the fixed regression coefficients up to the fourth polynomial degree of age ($A_{ij}$) for body weight and up to the fifth polynomial degree of age $\left(A_{ij}\right)$ for laying performance, $b_{sv}$ are the fixed regression coefficients of the interaction between genotype and age, $b_{tv}$ are the fixed regression coefficients of the interaction between diet and age, $b_{uv}$ are the fixed regression coefficients of the interaction between genotype, diet and age, $p_{k}$ is the random effect of the pen and $e_{ijkl}$ is the random error. For the polynomial analysis the MIXED procedure of SAS was used. Akaike’s information criterion (AIC) was used to determine the model with the best fit according to Koehn et al. [27]. Sample sizes for body weight are listed in the Supplementary Material (Table S1).

For the analysis of daily feed consumption, the experiment was split up into 3 periods (Period 1: week 18–30; Period 2: week 31–39; Period 3: week 40–51), because the variability of the daily feed use between the single weeks was rather high. The statistical model was similar to that described below for the egg quality traits (Equation (2). Results are presented in Supplementary Material S1 (Figure S1).

Data for egg weight, eggshell breaking strength and eggshell thickness were analyzed with a linear mixed model as following:

(2) $$Y_{ijklm}=\mu +G_{i}+D_{j}+A_{k}+G_{i}D_{j}+G_{i}A_{k}+D_{j}A_{k}+G_{i}D_{j}A_{k}+p_{l}+e_{ijklm}$$

where $Y_{ijklm}$ is the respective parameter, μ is the overall mean, $G_{i}$ is the fixed effect of the genotype (i = 1 to 3), $D_{j}$ is the fixed effect of the diet (j = 1 to 3), $A_{k}$ is the fixed effect of the age in weeks, $G_{i}D_{j}$, $G_{i}A_{k}$, $D_{j}A_{k}$ and $G_{i}D_{j}A_{k}$ are the interactions of the respective variables, $p_{l}$ is the random effect of the pen and $e_{\mathrm{ijklm}}$ is the random error.

As shell weight and egg weight are highly correlated [28], shell weight was calculated with and without including egg weight as a covariate in the analysis. The applied model was the same as described above for the other egg traits (Equation (2). To verify the correlation of egg weight and shell weight in the experimental data, Pearson’s correlation coefficients ($r_{p}$) between egg and shell weight were calculated. They were $r_{p}=\;$0.70 in experiment A and $r_{p}=\;$0.74 in experiment B. The calculation of the least squares means (LS-means) and testing of significant differences was carried out as described in Nolte et al. [29]. The calculation of daily feed intake and egg parameters was performed with the GLIMMIX procedure of SAS.

In the first experiment, an infestation with the northern fowl mite (*Ornithonyssus sylviarum*) took place in the barn and during this period a massive discrepancy in the data compared to the time before and after the infestation was realized. For that reason, the affected data from week 31–39 were excluded from the final analysis. In the case of body weight and laying performance a calculation with stepwise exclusion of data were applied to model the growth and laying curves. The full data, analyzed with a linear mixed model together with the final curves is presented in Supplementary Material Figures S2 (Body weight) and S3 (Laying performance).

The bone characteristics were analyzed using the GLIMMIX procedure of SAS according to the following model:

(3) $$Y_{ijkl}=\mu +G_{i}+D_{j}+G_{i}D_{j}+S_{k}+e_{ijkl}$$

where $Y_{ijkl}$ is the respective bone characteristic, μ is the overall mean, $G_{i}$ is the genotype (i = 1 to 3), $D_{j}$ is the diet (j = 1 to 3), $G_{i}D_{j}$ is their interaction, $S_{k}$ is the random effect of the sire and $e_{ijkl}$ is the random error. Since bone weight attributed a relatively large effect on bone strength [12], this factor was considered as a covariate for the analysis of bone breaking strength. Sample sizes for bone characteristics are listed in the Supplementary Material (Table S1).

## 3. Results

### 3.1. Hen Performance

**Figure 2:**
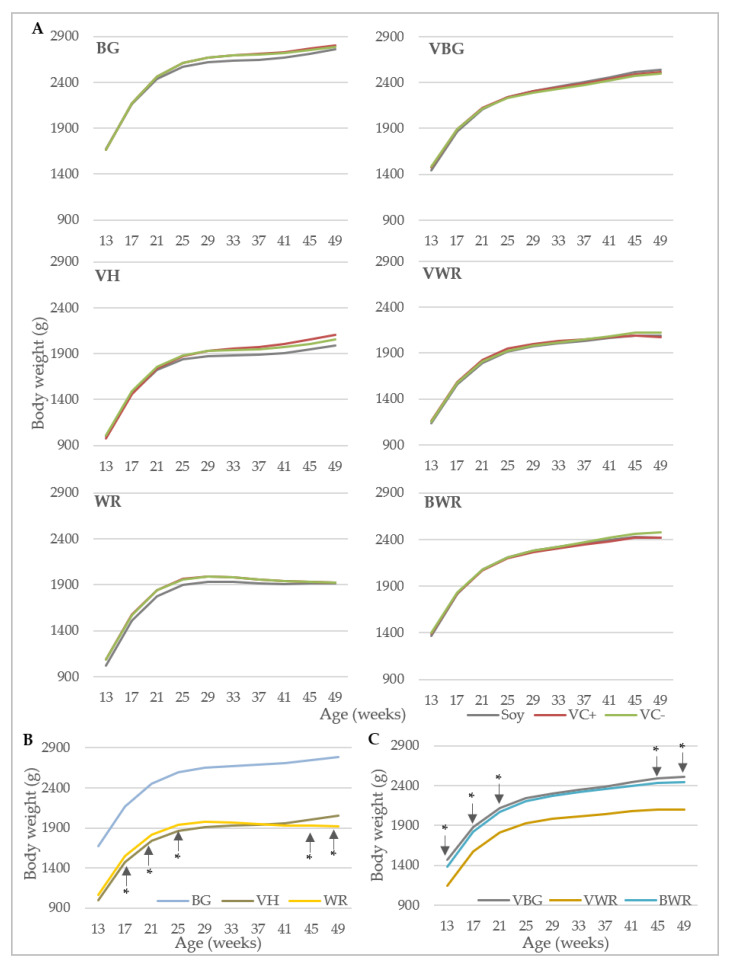
Body weight of hens. (**A**) Body weight development of six genotypes under the influence of different diets (BG, VH, WR: *n* = 38; VBG, VWR, BWR: *n* = 43). Within genotype and week, there were no significant differences between the feeding groups. (**B**) Comparison of body weight development of the purebreds (*n* = 113). (**C**) Comparison of body weight development of the crossbreds (*n* = 131). * mark significant differences between VH and WR (**B**) and VBG and BWR genotypes (**C**), respectively; in all other weeks the respective genotypes differ only significantly from BG (**B**) and VWR (**C**), respectively. BG: Bresse Gauloise, VH: Vorwerkhuhn, WR: White Rock, VBG: VH male × BG female, VWR: VH male × WR female, BWR: BG male × WR female.

The growth curves of the genotype x diet combinations and of the genotypes in comparison with each other are shown in Figure 2. In both experiments, there were no significant differences between the feeding groups within the genotypes (Figure 2A). Although the growth curves started to flatten between week 21 and 25, all hens except WR gained weight until the end of the experiment. The BG-crosses VBG and BWR reached final weights of almost 2.5 kg, which was about 300 g less than the BG. The VH and VWR achieved weights of ca. 2.0 kg, while the WR did not exceed 1.9 kg (Figure 2A). The comparison of the purebreds showed significant differences during the whole experiment with BG being significantly heavier than VH and WR. VH and WR differed statistically significantly from each other only in weeks 17–25 and 45–49 (Figure 2B), while in the first section WR were heavier than VH, this was opposite at the later section. In Figure 2C the equivalent analysis for the crossbreds is shown. In experiment B, the VWR hens have been significantly lighter than the other two genotypes during the whole experiment, while VBG and BWR only differed significantly in weeks 13–21 and 45–49, VBG being slightly heavier. Adjusted by the mean of age, no significant differences between the diets were found in both experiments.

**Figure 3:**
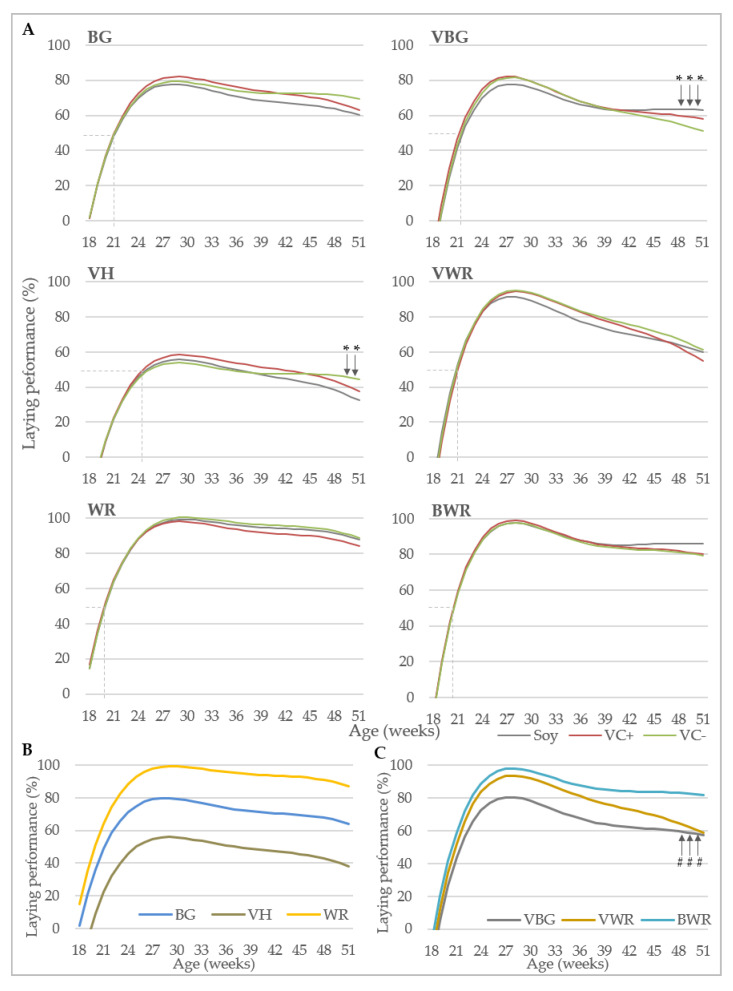
Laying performance. (**A**) Laying performance of six genotypes under the influence of different diets (*n* = 2). Dashed lines indicate the age at 50% egg production. * mark significant differences between soy and VC− groups (*p* < 0.05). (**B**) Comparison of laying performance of purebreds (*n* = 6). All genotypes differ significant from each other in every week at *p* < 0.05. (**C**) Comparison of laying performance of crossbreds (*n* = 6). # indicates that there is no significant difference between VBG and VWR in the respective weeks; at all other time points, all genotypes differ significantly at *p* < 0.05. BG: Bresse Gauloise, VH: Vorwerkhuhn, WR: White Rock, VBG: VH male x BG female, VWR: VH male × WR female, BWR: BG male × WR female.

Figure 3 illustrates the laying performance over the course of the experimental period. In Figure 3A, the comparison of the feeding groups within genotypes is shown. In BG and WR no significant differences between the different diets were found, while in VH in the last two weeks of the experiment the difference between the VC− and the soy group became statistically significant with a difference of −5.56% and −12.02% in weeks 50 and 51, respectively. A significant difference between the soy and VC− group was also found in experiment B in VBG at the end of the experiment, but here the soy group showed a significantly higher laying performance in weeks 49–51 compared to the VC− group of 5.41–6.97%. In VWR and BWR no significant differences in the laying curve between the feeding groups could be detected.

The WR and BWR groups showed the highest peak production of about 100% in the respective experiments (Figure 3B,C). Among all genotypes, the VH hens showed the lowest laying performance, which is reflected in a later laying maturity and an overall peak production of only about 56%. The laying performance of BG was in between the WR and VH, all purebreds differed significantly from each other at all time points of the experiment (Figure 3B). VWR hens performed similar as the BWR but were behind in terms of laying persistency (Figure 3C). The laying performance of VBG was lower than that of BWR and VWR, but comparable to that of BG in experiment A regarding laying maturity and peak production. The crossbred genotypes differed significantly from each other from week 18–48, but in weeks 49–51 the difference between VWR and VBG became smaller and was not statistically significant anymore, due to a low persistency of the VWR. Regarding laying maturity, VH hens reached 50% egg production only at week 25, while the other genotypes exceeded this threshold between 20 and 22 weeks. All crossbred genotypes reached peak egg production one week earlier than the purebreds, namely at week 28. No significant differences regarding laying performance could be detected between the feeding groups in both experiments.

Regarding daily feed consumption, significant differences were only detected in experiment B (Figure S1). In period 3, in cross VBG a significant difference was found between the soy and VC+ group, with the soy group consuming almost 40 g of feed more per day. Regarding the main factor genotype, a significant difference of 10.8 g existed between the crossbreds VBG and VWR.

### 3.2. Egg Parameters

The effects of genotype, diet, age and their interactions on egg weight, shell breaking strength, shell weight and thickness are shown in Table 2. In both experiments, the egg quality was significantly influenced by genotype and age. While for the purebreds, a dietary effect was only accounted for egg weight and shell weight, all egg quality traits were significantly influenced by the diet in the crossbreds.

**Figure 4:**
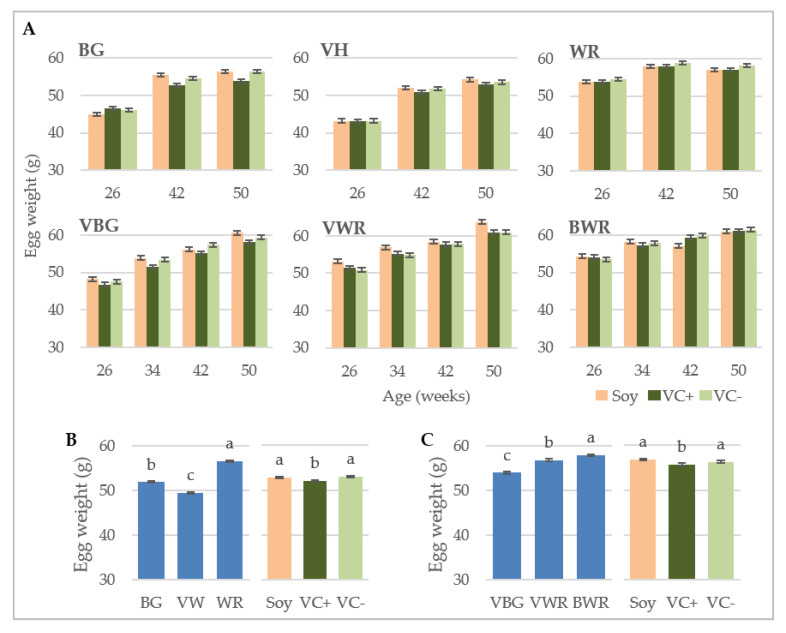
Egg weight. LS-means ± SE. (**A**) Effect of diet on egg weight (*n* = 96). (**B**) Purebreds: Mean egg weights of the respective genotypes and diets (*n* = 288). (**C**) Crossbreds: Mean egg weights of the respective genotypes and diets (*n* = 288). BG: Bresse Gauloise, VH: Vorwerkhuhn, WR: White Rock, VBG: VH male × BG female, VWR: VH male x WR female, BWR: BG male × WR female. ^a,b,c^ Bars differ significantly at *p* < 0.05.

Figure 4A shows the LS-mean values for egg weight of the different genotypes at different measurements. Figure 4B,C illustrate the LS-mean values for the main factors diet and genotype over all measurements. In all genotypes the egg weight increased with aging of the hens, only WR showed 1 g lighter eggs in week 50 than in week 42 (Figure 4A). In case of the purebreds the mean egg weight was highest for WR (56.6 g) and lowest for VH (49.5 g) (Figure 4B), while among the crossbreds BWR laid the heaviest eggs (58.0 g) and the VBG the lightest (54.0 g). In both experiments, the egg weights of all genotypes differed significantly from each other, while within genotypes and measurements no significant differences between the feeding groups were detected (Figure 4A). Over the whole experiment, the VC+ groups had significantly lighter eggs than the soy and VC− groups, which was true in the pure- (Figure 4B) and crossbreds (Figure 4C). However, these differences were not pronounced and amounted to 1.1 g or even less.

Mean values of eggshell breaking strength and eggshell thickness of the genotypes and diets are presented in Table 3 and Table 4 for pure- and crossbreds, respectively. The shell strength was significantly highest for WR (57.68 N) and VWR (58.41 N) in experiments A and B, respectively, while purebred BG and VH hens showed values lower than 50 N. Differences between the dietary groups were only found in the crossbreds where the VC+ group showed a significantly reduced eggshell breaking strength. The same holds true for the shell thickness.

The results of the statistical analysis of the shell weight with and without consideration of the egg weight as a covariate in the model are shown in Table 3 and Table 4. In the purebreds, there was no significant interaction between diet and egg weight and consequently the values changed due to the correction but not the significances. Nevertheless, the shell weight of the local breeds was increased as a result of the correction factor, but in both applied models the WR group exhibited a significantly higher shell weight than the local breeds. A similar situation was found in experiment B, where the cross of the two local breeds, VBG, showed significantly lower shell weights than the other genotypes, even after the covariate was considered in the statistical model. With regard to the effect of the diet, the correction eliminated the significant differences in favor of the VC+ group in Experiment A, which was present without the consideration of the egg weight as a correction factor in the model. In the second experiment (B) there were significant differences between all feeding groups with the soy group having the highest shell weights and the VC+ group the lowest and this was the case with and without the covariate egg weight.

### 3.3. Bone Characteristics

The effects of genotype, diet and their interaction on the bone characteristics within the purebreds and crossbreds are shown in Table 5. In both experiments, the genotype had a highly significant effect on all bone traits. In contrast, the diet influenced only the bone mineral density of the tibiotarsus and the keel bone in the purebreds. The genotype by diet interaction was not significant at all. In both bone types, the breaking strength was significantly influenced by considering bone weight in the statistical model.

The corresponding LS-means of the purebreds are listed in Table 6. Group VH showed the highest tibiotarsus breaking strength, followed by group BG and WR. In the case of the humerus, group BG exhibited a higher breaking strength compared to the VH group. The WR group showed the lowest breaking strength for this bone type as well. The significantly highest bone mineral density in all three bones was observed in BG hens, followed by VH and WR. The BG group had the significantly heaviest and longest bones. For tibiotarsus thickness, the highest values were found in VH hens, while in humerus the WR group was inferior to the other genotypes. With exception of the humerus weight, the WR hens showed the lowest values in all bone characteristics among the purebred genotypes. Regarding the effect of the diet, the VC+ group significantly differed from the controls (Soy) showing a higher bone mineral density for both the tibiotarsus and keel bone, whereas the VC− group was intermediate.

Table 7 shows the results of bone characteristics of the crossbreds. The significantly highest breaking strength and bone mineral density in all bone types was observed in VBG hens. Group VWR had the significantly lowest keel bone mineral density, while the other two groups did not differ from each other. The groups VWR and BWR both had a significantly higher cortical area than group VBG. The BG crosses had significantly higher values for tibiotarsus weight and length, while in the humerus it was more differentiated. The latter also applies to the bone thickness in both bone types. The diet had no effect on the crossbreds.

## 4. Discussion

In the two experiments of this study, hens of different genotypes were fed diets with 20% VC-rich and VC-poor faba beans. In general, the chicken genotype had more impact on the parameters measured than the different diets.

### 4.1. Comparison of Genotypes

The body weight is a breed characteristic defined in the breed standard. For both Vorwerkhuhn and Bresse Gauloise hens, it is 2000–2500 g [30,31], which matches with the final weight of the VH hens, while the BG in the present study have been clearly heavier with more than 2700 g. Lambertz et al. [24] recorded weights of 2957 g for BG hens slaughtered at 75 weeks of age. The weight of the WR hens is in the range indicated for commercial brown laying hybrids [32,33]. Despite the differences in body weight, all purebreds had a similar feed consumption. In the case of BG and WR, this is assumed to be due the high performance either regarding growth (BG) or laying (WR). For VH possible explanations are an unfavorably high metabolic rate or feed wastage by foraging. The latter probably also applies to the VWR group in experiment B.

The laying performance of the local breeds and especially of the VH has been considerably lower than that of the WR. Similar differences between local and commercial genotypes have also been described by Lange [34] and Götze and von Lengerken [4], who investigated both the laying performance of several German local breeds. The described difference is due to the different breeding history of these breeds. While commercial laying hybrids have been intensively selected for high number of saleable eggs for many generations as part of the breeding program [35], the local breeds were typically presented on exhibitions and therefore the type was the most important trait in the last decades. The performance divergence is also reflected by the laying maturity and age at 50% egg production. The persistency of all three genotypes was similar in relation to their difference in total laying performance, as indicated by the almost parallel course of the laying curves.

Furthermore, the egg weights of the local breeds have been lower than of WR, which is in agreement with Sirri et al. [36] and Moula et al. [28], who compared the performance of commercial laying hybrids with Italian and Belgian local breeds, respectively. Lambertz et al. [24] described a high amount of small eggs (<53 g) at the beginning of the laying period of BG, whereas at the end of the laying period small eggs amounted only 3%.

With regard to the eggshell quality, i.e., breaking strength, thickness and weight, there was also a clear difference between the commercial WR line and the local breeds BG and VH in the present study, whereas Moula et al. [28] did not find this difference in breaking strength, but also in shell weight and thickness. Tixier-Boichard et al. [37] and Götze and von Lengerken [4] also found no differences in the breaking strength of eggshells of local versus commercial genotypes.

The laying performance of the crossbreds was in the first half of the laying period more similar to the performance level of the maternal genotype than to the paternal, while in the second half, there was a decrease in direction of the paternal performance, although BWR showed a much better persistency than VWR in their laying performance. The mean egg weights of the crossbreds have been higher than the mean of the parental pure lines for all genotypes, which, however cannot be construed directly as heterosis, since the difference might be confounded with a year effect. Concerning the eggshell parameters, the crossbreds’ values have been in between that of the parental lines.

Consistent with the literature [38], this research revealed considerable phenotypic differences in terms of bone traits between the genotypes. Our findings support the hypothesis that bone morphometry has only limited influence on bone breaking strength [9]. The results suggest the crossbreds being heterotic, as they showed enhanced values in comparison with the respective purebred parents [39]. This is especially true for the BG crosses. However, this must be interpreted with caution, as the experiments were conducted separately and a direct comparison is not possible. Another source of uncertainty is that the results are somewhat contradictory. In the case of the breaking strength, possible hybrid vigor occurred in the humerus but not in the tibiotarsus. In terms of bone mineral density, it was the opposite. However, this result cannot be conclusively clarified based on the available data but suggests more specifically designed follow-up studies.

### 4.2. Comparison of Diets

Regarding the hens’ body weight, no influence of faba bean feeding on the weight development was observed, which is in accordance with previous reports [21,40], where laying hens were fed 25% or 24% faba beans and no effect on the laying performance was reported. In contrast, however, Halle [17] observed performance reductions already at a faba bean level of 10% and Fru-Nji et al. [18] from a level of 16% faba beans and higher. These differences might be explained by the faba beans that have been used in the respective studies, because the VC content differs between varieties [41] and the percentage of faba beans in the diet gives no direct information about the VC content.

The reduction of egg-weight with a faba-bean diet was shown in several studies [17,21,42] with an extent of 2–4 g per egg, therefore vicin has also been known as ‘egg-weight-depressing factor’ since its identification in faba beans forty years ago [19]. Egg-weight reduction was also evident in the VC+ groups in the present study but the difference amounted to less than 1 g compared to control and VC− groups.

Regarding eggshell stability, no influence of faba beans on breaking strength or shell thickness was observed by other authors [40,43], which is in accordance with the results of experiment A, whereas both parameters have been reduced in the VC+ groups of experiment B.

To our knowledge, this is the first trial studying the effects of VC on bone characteristics in laying hens. Although the VC+ group showed a significantly higher bone mineral density than the soy group, this difference is still small. This especially applies to the keel bone, where the p-value was only slightly below the critical threshold. Furthermore, no effects on the humerus were observed, although the bones of the purebreds otherwise showed a very consistent pattern. Thus, a distinct influence of VC on the bones cannot be confirmed. A negative effect of tannin on bone development, as demonstrated in broilers [44,45], is also considered unlikely, as the tannin content fluctuated only marginally between diets and the VC+ diet even had the lowest value.

## 5. Conclusions

Taken together the dietary effect, only little negative impact of VC was observed. However, as it concerned mainly the economically important parameter egg weight, the VC− diet should be preferred when replacing soy with regional faba beans.

With regard to the genotype, the commercial WR line was superior in performance parameters but characterized by inferior bone stability compared to the local breeds. Although no direct comparison of the two experiments is possible, the findings suggest that the crossbreeding with the meat-type BG improved the bone characteristics of WR with almost equal laying performance.

Because this study is dealing with dual-purpose genotypes, the male performance also has to be considered for a final conclusion. The performance test of the males showed BG to improve the fattening performance of the layer-type VH and WR chickens [29]. Therefore, considering both sexes, the BWR hybrid seems to be the most promising cross. However, further research is needed to characterize crossbreeding as a possibility for agricultural use of local chicken breeds. Moreover, the inner egg quality is a topic to be discussed, which is underway in a follow-up study.

## Figures and Tables

**Table 1:** Composition, analyzed and calculated nutrient composition of the experimental diets.

Item	Experiment A			Experiment B		
	Soy	VC+	VC−	Soy	VC+	VC−
Ingredients (%)						
Wheat	40.39	29.78	29.78	40.39	29.78	29.78
Corn	10.00	10.89	10.89	10.00	10.89	10.89
Soybean meal (39.8% CP)	11.84	-	-	11.84	-	-
Blue sweet lupine cv. Boruta	21.00	21.13	21.13	21.00	21.13	21.13
Faba bean cv. Fuego	-	20.00	-	-	20.00	-
Faba bean cv. Tiffany	-	-	20.00	-	-	20.00
Soybean oil	4.00	5.77	5.77	4.00	5.77	5.77
Dicalcium phosphate	2.76	2.51	2.51	2.76	2.51	2.51
Calcium carbonate	8.39	8.25	8.25	8.39	8.25	8.25
Sodium chloride	0.32	0.25	0.25	0.32	0.25	0.25
DL-Methionine	0.21	0.28	0.28	0.21	0.28	0.28
Lysine	0.09	0.10	0.10	0.09	0.10	0.10
Tryptophan	-	0.04	0.04	-	0.04	0.04
Premix ^1^	1.00	1.00	1.00	1.00	1.00	1.00
Chemical composition						
Dry matter abs (%) ^2^	89.60	89.50	89.50	90.90	91.20	90.90
Crude ash (g/kg DM) ^2^	152.60	149.30	148.70	129.70	139.50	146.70
Crude protein (g/kg DM) ^2^	182.40	171.90	185.30	185.20	202.10	184.10
Crude fat (g/kg DM) ^2^	95.00	88.80	97.40	91.10	83.70	91.80
Crude fiber (g/kg DM) ^2^	54.60	50.30	54.50	61.80	51.30	59.20
Starch (g/kg DM) ^2^	362.60	393.10	349.50	365.90	349.5	347.00
Sucrose (g/kg DM) ^2^	29.70	25.00	24.00	24.60	26.60	24.60
SFA (g/100g fat) ^2^	17.70	17.00	16.10	17.6	16.80	16.40
MUFA (g/100g fat) ^2^	22.50	22.60	22.80	22.70	22.80	21.80
PUFA (g/100g fat) ^2^	59.80	60.40	61.10	59.6	60.40	61.70
Vicine (%) ^2^	0.016	0.079	0.003	0.0	0.095	0.015
Convicine (%) ^2^	0.006	0.037	0.002	0.0	0.039	0.004
VC (Vicin + Convicin; %) ^3^	0.022	0.116	0.005	0.0	0.134	0.019
Tannin (mg/g) ^2^	3.51	3.02	3.33	3.22	3.91	3.67
AMEn (MJ/kg) ^3,4^	12.53	12.60	12.36	12.43	12.19	12.12
Methionine (%) ^3^	0.42	0.44	0.44	0.42	0.44	0.44
Lysine (%) ^3^	0.81	0.83	0.83	0.81	0.83	0.83
Tryptophan (%) ^3^	0.16	0.17	0.17	0.16	0.17	0.17
Threonine (%) ^3^	0.58	0.55	0.55	0.58	0.55	0.55

CP: crude protein, SFA: saturated fatty acids, MUFA: monounsaturated fatty acids, PUFA: polyunsaturated fatty acids, AME_n_: nitrogen-corrected apparent metabolizable energy; ^1^ Premix-hens: feed additives (per kg premix): Vitamin A, 1,000,000 IU; Vitamin D3, 250,000 IU; Vitamin E, 2000 mg; Vitamin B1, 250 mg; Vitamin B2, 700 mg; Vitamin B6, 400 mg; Vitamin B12, 2000 μg; Vitamin K3, 400 mg; Nicotin amide, 4000 mg; Calcium-D-pantothenate, 1000 mg; Folic acid, 60 mg; Biotin, 2500 μg; Choline chloride, 40,000 mg; Fe, 4000 mg; Cu, 1000 mg; Mn, 10,000 mg; Zn, 8000 mg; I, 120 mg; Se, 25 mg; Co, 20.5 mg; Butylated hydroxy toluene (BHT), 12,500 mg; Beta-carotene, 400 mg; Canthaxanthin, 400 mg; ^2^ Analyzed; ^3^ Calculated; ^4^ Apparent metabolizable energy concentrations corrected to zero nitrogen balance (AMEn), calculated according to the energy estimation equation of the World’s Poultry Association (Vogt, 1986).

**Table 2:** Effect of genotype, diet, age and their interactions on different egg quality parameters.

Parameter	Statistics	Purebreds							Crossbreds						
		Genotype	Diet	Age	Genotype × Diet	Genotype × Age	Diet × Age	Genotype × Diet × Age	Genotype	Diet	Age	Genotype × Diet	Genotype × Age	Diet × Age	Genotype × Diet × Age
Egg weight	F value	357.36	2.32	947.31	1.33	68.39	4.62	2.38	59.95	4.26	834.78	3.18	21.75	6.59	0.77
	*p* value	<0.0001	0.0018	<0.0001	0.2576	<0.0001	0.0010	0.0149	<0.0001	0.0143	<0.0001	0.0129	<0.0001	<0.0001	0.6820
Shell breaking strength	F value	50.97	2.61	10.44	0.17	5.57	1.94	2.22	52.47	11.53	35.48	0.89	18.57	1.74	1.65
	*p* value	<0.0001	0.0736	<0.0001	0.9543	0.0002	0.1019	0.0233	<0.0001	<0.0001	<0.0001	0.4711	<0.0001	0.1079	0.0705
Shell weight	F value	251.58	4.42	44.30	0.08	31.12	4.91	3.37	65.96	18.96	291.10	1.93	20.70	2.13	2.18
	*p* value	<0.0001	0.0121	<0.0001	0.9881	<0.0001	0.0006	0.0008	<0.0001	<0.0001	<0.0001	0.1026	<0.0001	0.0472	0.0103
Shell weight adj.*	F value	105.11	2.26	91.72	0.23	3.36	2.97	2.85	24.75	22.97	04.11	1.52	5.22	2.36	2.56
	*p* value	<0.0001	0.1044	<0.0001	0.9239	0.0094	0.0185	0.0037	<0.0001	<0.0001	0.0064	0.1926	<0.0001	0.0284	0.0022
Shell thickness	F value	102.07	2.03	260.47	0.05	8.72	0.79	2.20	70.92	10.16	135.59	0.32	27.11	2.55	3.05
	*p* value	<0.0001	0.1314	<0.0001	0.9950	<0.0001	0.5331	0.0246	<0.0001	<0.0001	<0.0001	0.9750	<0.0001	0.0180	<0.0001

* Shell weight analyzed with egg weight as co-variable, *p* values for egg weight have been *p* < 0.0001 in pure- and crossbreds.

**Table 3:** LS-means ± SE for eggshell breaking strength, shell thickness and shell weight in purebreds (experiment A).

Parameter	Genotype			Diet		
	BG	VH	WR	Soy	VC+	VC−
Shell breaking strength (N)	44.71 ^b^ ± 0.95	47.58 ^b^ ± 0.96	57.68 ^a^ ± 0.95	51.22 ^a^ ± 0.96	48.26 ^a^ ± 0.95	50.50 ^a^ ± 0.95
Shell thickness (mm)	0.33 ^b^ ± 0.003	0.32 ^c^ ± 0.003	0.38 ^a^ ± 0.003	0.35 ^a^ ± 0.003	0.34 ^a^ ± 0.003	0.34 ^a^ ± 0.003
Shell weight (g)	5.09 ^b^ ± 0.05	4.74 ^c^ ± 0.05	6.13 ^a^ ± 0.05	5.39 ^a^ ± 0.05	5.21 ^b^ ± 0.05	5.37 ^a^ ± 0.05
Shell weight adj * (g)	5.17 ^b^ ± 0.04	5.01 ^c^ ± 0.04	5.85 ^a^ ± 0.04	5.40 ^a^ ± 0.04	5.27 ^a^ ± 0.04	5.36 ^a^ ± 0.04

* Shell weight analyzed with egg weight as co-variable (*n* = 288). ^a,b,c^ Values not sharing a letter within row and category differ significantly at *p* < 0.05.

**Table 4:** LS-means ± SE for eggshell breaking strength, shell thickness and shell weight in crossbreds (experiment B).

Parameter	Genotype			Diet		
	VBG	VWR	BWR	Soy	VC+	VC−
Shell breaking strength (N)	53.61 ^b^ ± 0.52	58.41 ^a^ ± 0.52	50.96 ^c^ ± 0.52	56.06 ^a^ ± 0.52	52.52 ^b^ ± 0.52	54.40 ^a^ ± 0.52
Shell thickness (mm)	0.33 ^c^ ± 0.002	0.35 ^b^ ± 0.002	0.36 ^a^ ± 0.002	0.35 ^a^ ± 0.002	0.34 ^b^ ± 0.002	0.35 ^a^ ± 0.002
Shell weight (g)	5.28 ^b^ ± 0.03	5.65 ^a^ ± 0.03	5.70 ^a^ ± 0.03	5.67 ^a^ ± 0.03	5.43 ^c^ ± 0.03	5.53 ^b^ ± 0.03
Shell weight adj* (g)	5.44 ^b^ ± 0.02	5.61 ^a^ ± 0.02	5.57 ^a^ ± 0.02	5.63 ^a^ ± 0.02	5.47 ^c^ ± 0.02	5.53 ^b^ ± 0.02

* Shell weight analyzed with egg weight as co-variable (*n* = 288). ^a,b,c^ Values not sharing a letter within row and category differ significantly at *p* < 0.05.

**Table 5:** Effect of genotype, diet and their interaction on bone characteristics of tibiotarsus, humerus and keel bone.

Bone Type	Parameter	Statistics	Purebreds				Crossbreds			
			Genotype	Diet	Genotype × Diet	Bone Weight	Genotype	Diet	Genotype × Diet	Bone Weight
Tibiotarsus	Breaking strength	F value	333.01	0.64	1.44	297.77	219.91	1.60	1.25	312.87
		*p* value	<0.0001	0.5274	0.2213	<0.0001	<0.0001	0.2038	0.2905	<0.0001
	Mineral density	F value	152.82	5.24	0.42	---	44.14	0.38	0.50	---
		*p* value	<0.0001	0.0058	0.7967	---	<0.0001	0.6869	0.7388	---
	Cortical area	F value	102.62	1.62	0.67	---	46.19	1.25	1.54	---
		*p* value	<0.0001	0.2002	0.6154	---	<0.0001	0.2863	0.1912	---
	Weight	F value	109.36	2.87	0.13	---	27.50	0.40	0.79	---
		*p* value	<0.0001	0.0580	0.9725	---	<0.0001	0.6730	0.5292	---
	Length	F value	144.58	1.15	0.92	---	20.11	0.08	0.29	---
		*p* value	<0.0001	0.3193	0.4534	---	<0.0001	0.9250	0.8863	---
	Thickness	F value	15.58	1.84	1.01	---	3.57	0.16	0.72	---
		*p* value	<0.0001	0.1604	0.3998	---	0.0292	0.8502	0.5788	---
Humerus	Breaking strength	F value	255.24	0.37	1.52	223.51	23.45	0.53	0.13	332.89
		*p* value	<0.0001	0.6880	0.1972	<0.0001	<0.0001	0.5893	0.9731	<0.0001
	Mineral density	F value	110.86	0.50	0.03	---	58.36	1.49	1.36	---
		*p* value	<0.0001	0.6043	0.9977	---	<0.0001	0.2273	0.2468	---
	Weight	F value	61.14	0.30	0.04	---	29.63	1.26	0.68	---
		*p* value	<0.0001	0.7431	0.9966	---	<0.0001	0.2848	0.6093	---
	Length	F value	50.95	0.38	0.80	---	7.83	0.31	1.00	---
		*p* value	<0.0001	0.6872	0.5248	---	0.0005	0.7313	0.4092	---
	Thickness	F value	15.81	2.36	0.68	---	3.26	0.12	0.13	---
		*p* value	<0.0001	0.0962	0.6082	---	0.0394	0.8839	0.9715	---
Keel bone	Mineral density	F value	61.52	3.05	0.31	---	16.33	1.19	0.96	---
		*p* value	<0.0001	0.0489	0.8710	---	<0.0001	0.3058	0.4282	---

**Table 6:** LS-means ± SE for characteristics of tibiotarsus, humerus and keel bone under the effect of genotype and diet for experiment A (purebreds) (*n* = 113).

Bone Type	Parameter	Genotype			Diet		
		BG	VH	WR	Soy	VC+	VC−
Tibiotarsus	Breaking strength (N)	274.87 ^b^ ± 4.13	321.81 ^a^ ± 3.36	197.23 ^c^ ± 3.67	261.61 ± 3.41	265.38 ± 3.32	266.93 ± 3.41
	Mineral density (g/cm^2^)	0.382 ^a^ ± 0.004	0.347 ^b^ ± 0.004	0.279 ^c^ ± 0.004	0.326 ^b^ ± 0.004	0.345 ^a^ ± 0.004	0.338 ^a,b^ ± 0.004
	Cortical area (%)	47.63 ^a^ ± 0.67	46.30 ^a^ ± 0.64	35.78 ^b^ ± 0.63	44.18 ± 0.65	42.70 ± 0.63	42.83 ± 0.65
	Weight (g)	14.52 ^a^ ± 0.14	12.54 ^b^ ± 0.14	11.68 ^c^ ± 0.13	12.67 ± 0.14	13.14 ± 0.13	12.93 ± 0.14
	Length (mm)	127.69 ^a^ ± 0.40	123.03 ^b^ ± 0.38	118.44 ^c^ ± 0.37	122.58 ± 0.39	123.27 ± 0.37	123.32 ± 0.39
	Thickness (mm)	6.34 ^b^ ± 0.03	6.57 ^a^ ± 0.03	6.39 ^b^ ± 0.03	6.39 ± 0.03	6.48 ± 0.03	6.43 ± 0.03
Humerus	Breaking strength (N)	359.06 ^a^ ± 4.68	327.82 ^b^ ± 4.21	230.15 ^c^ ± 4.03	308.56 ± 4.10	304.40 ± 3.98	304.06 ± 4.11
	Mineral density (g/cm^2^)	0.272 ^a^ ± 0.003	0.229 ^b^ ± 0.003	0.218 ^c^ ± 0.003	0.237 ± 0.003	0.241 ± 0.003	0.240 ± 0.003
	Weight (g)	6.96 ^a^ ± 0.09	5.63 ^b^ ± 0.09	5.89 ^b^ ± 0.09	6.11 ± 0.09	6.20 ± 0.09	6.18 ± 0.09
	Length (mm)	81.73 ^a^ ± 0.23	79.85 ^b^ ± 0.22	78.47 ^c^ ± 0.22	79.86 ± 0.23	80.07 ± 0.22	80.12 ± 0.23
	Thickness (mm)	5.86 ^a^ ± 0.03	5.88 ^a^ ± 0.03	5.68 ^b^ ± 0.03	5.78 ± 0.03	5.84 ± 0.03	5.82 ± 0.03
Keel bone	Mineral density (g/cm^2^)	0.222 ^a^ ± 0.002	0.195 ^b^ ± 0.002	0.187 ^c^ ± 0.002	0.197 ^b^ ± 0.002	0.205 ^a^ ± 0.002	0.202 ^a,b^ ± 0.002

^a,b,c^ Values not sharing a letter within bone trait and category differ significantly at *p* < 0.05.

**Table 7:** LS-means ± SE for characteristics of tibiotarsus, humerus and keel bone under the effect of genotype and diet for experiment B (crossbreds) (*n* = 131).

Bone Type	Parameter	Genotype			Diet		
		VBG	VWR	BWR	Soy	VC+	VC−
Tibiotarsus	Breaking strength (N)	297.65 ^a^ ± 2.92	244.91 ^b^ ± 3.01	227.16 ^c^ ± 2.92	255.55 ± 2.89	254.65 ± 2.89	259.52 ± 2.88
	Mineral density (g/cm^2^)	0.379 ^a^ ± 0.004	0.328 ^b^ ± 0.004	0.339 ^b^ ± 0.004	0.346 ± 0.004	0.350 ± 0.004	0.349 ± 0.004
	Cortical area (%)	55.42 ^b^ ± 0.43	60.09 ^a^ ± 0.43	60.75 ^a^ ± 0.43	58.42 ± 0.43	59.30 ± 0.43	58.54 ± 0.43
	Weight (g)	14.12 ^a^ ± 0.14	12.85 ^b^ ± 0.14	14.06 ^a^ ± 0.14	13.58 ± 0.14	13.72 ± 0.14	13.73 ± 0.14
	Length (mm)	126.37 ^a^ ± 0.37	123.95 ^b^ ± 0.37	127.15 ^a^ ± 0.37	125.89 ± 0.37	125.70 ± 0.37	125.87 ± 0.37
	Thickness (mm)	6.42 ^b^ ± 0.03	6.48 ^a,b^ ± 0.03	6.54 ^a^ ± 0.03	6.47 ± 0.03	6.50 ± 0.03	6.48 ± 0.03
Humerus	Breaking strength (N)	350.65 ^a^ ± 4.47	305.89 ^c^ ± 4.60	335.58 ^b^ ± 4.38	333.65 ± 4.43	327.30 ± 4.36	331.16 ± 4.36
	Mineral density (g/cm^2^)	0.263 ^a^ ± 0.003	0.223 ^c^ ± 0.003	0.243 ^b^ ± 0.003	0.246 ± 0.003	0.239 ± 0.003	0.244 ± 0.003
	Weight (g)	7.15 ^a^ ± 0.10	6.12 ^c^ ± 0.10	6.82 ^b^ ± 0.10	6.77 ± 0.10	6.57 ± 0.10	6.75 ± 0.10
	Length (mm)	81.64 ^a,b^ ± 0.22	81.00 ^b^ ± 0.22	82.23 ^a^ ± 0.22	81.53 ± 0.22	81.76 ± 0.22	81.57 ± 0.22
	Thickness (mm)	5.99 ^a^ ± 0.03	5.89 ^b^ ± 0.03	5.94 ^a,b^ ± 0.03	5.94 ± 0.03	5.95 ± 0.03	5.93 ± 0.03
Keel bone	Mineral density (g/cm^2^)	0.221 ^a^ ± 0.002	0.204^b^ ± 0.002	0.214 ^a^ ± 0.002	0.210 ± 0.002	0.214 ± 0.002	0.214 ± 0.002

^a,b,c^ Values not sharing a letter within bone trait and category differ significantly at *p* < 0.05.

## Supplementary Materials

The following are available online at https://www.mdpi.com/2076-2615/10/9/1480/s1, Table S1: Sample sizes of the analysis separated by genotype x diet combination; Figure S1: Daily feed consumption; Figure S2: Modeling body weight of hens; Figure S3: Modeling of laying performance.
