# Supplementary material for: Egg Production and Bone Stability of Local Chicken Breeds and Their Crosses Fed with Faba Beans
Source: Animals (Basel). 2020 Aug 22;10(9):1480. doi: 10.3390/ani10091480 (PMC7552325; doi:10.3390/ani10091480)
Supplement: Supplementary file 1 [file animals-10-01480-s001.zip › Supplement_FigureS1.pdf]

Supplementary Material

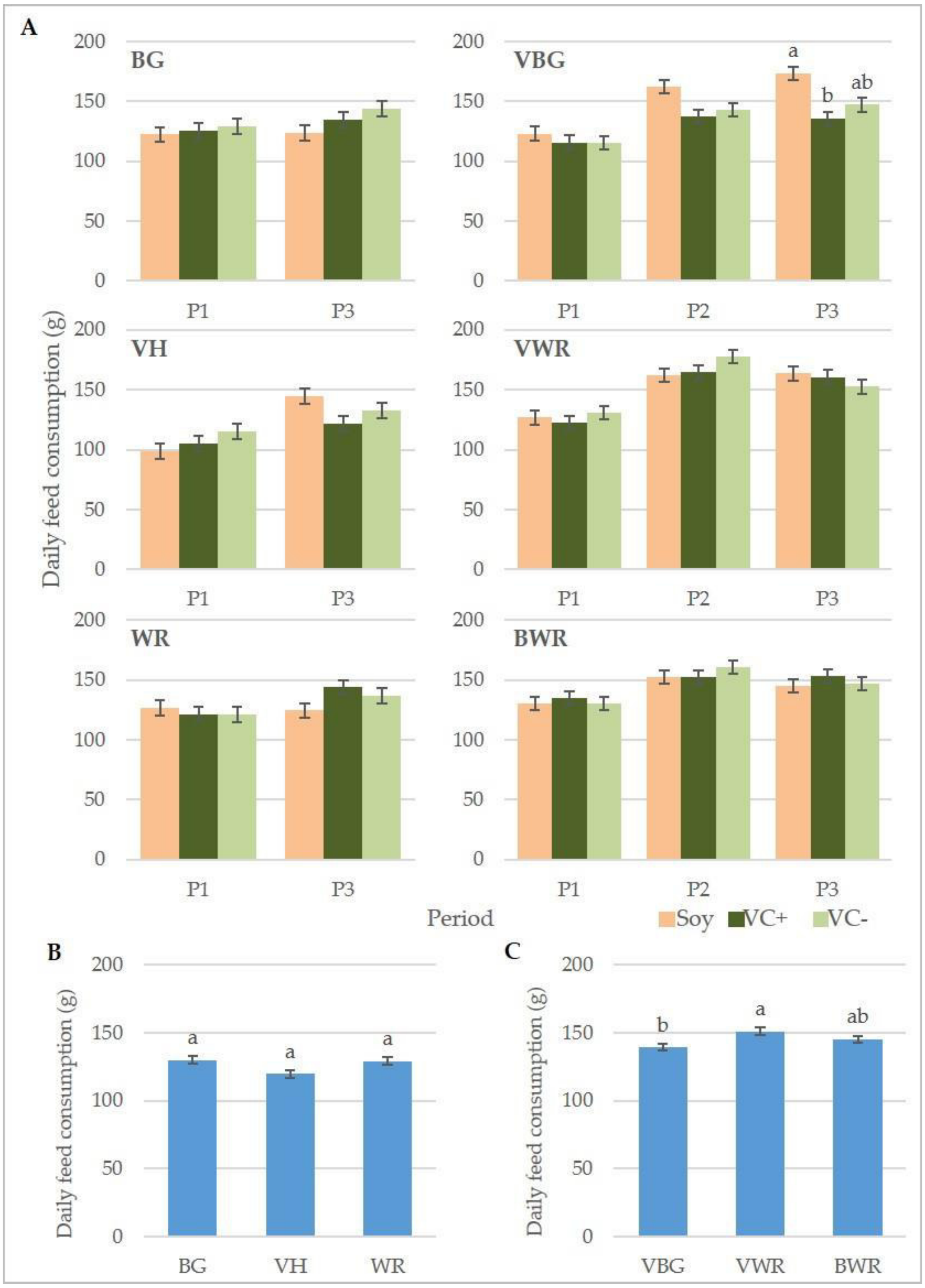

Figure S1. Daily feed consumption, LS-means ± SE. **(A)** Daily feed consumption of six genotypes under the influence of different diets during three periods. Period 1 from week 18-30, period 2 from week 31-39, period 3 from week 40-51. **(B)** Daily feed consumption of the respective purebreds. **(C)** Daily feed consumption of the respective crossbreds. BG: Bresse Gauloise, VH: Vorwerkhuhn, WR: White Rock, VBG: VH male x BG female, VWR: VH male x WR female, BWR: BG male x WR female. [a,b] Bars not sharing a letter differ at $p < 0.05$. In (A) significant differences were only shown within genotype and period. Groupings without superscripts shown no significant differences.
