# Supplementary material for: Egg Production and Bone Stability of Local Chicken Breeds and Their Crosses Fed with Faba Beans
Source: Animals (Basel). 2020 Aug 22;10(9):1480. doi: 10.3390/ani10091480 (PMC7552325; doi:10.3390/ani10091480)
Supplement: Supplementary file 1 [file animals-10-01480-s001.zip › Supplement_FigureS2.pdf]

Supplementary Material

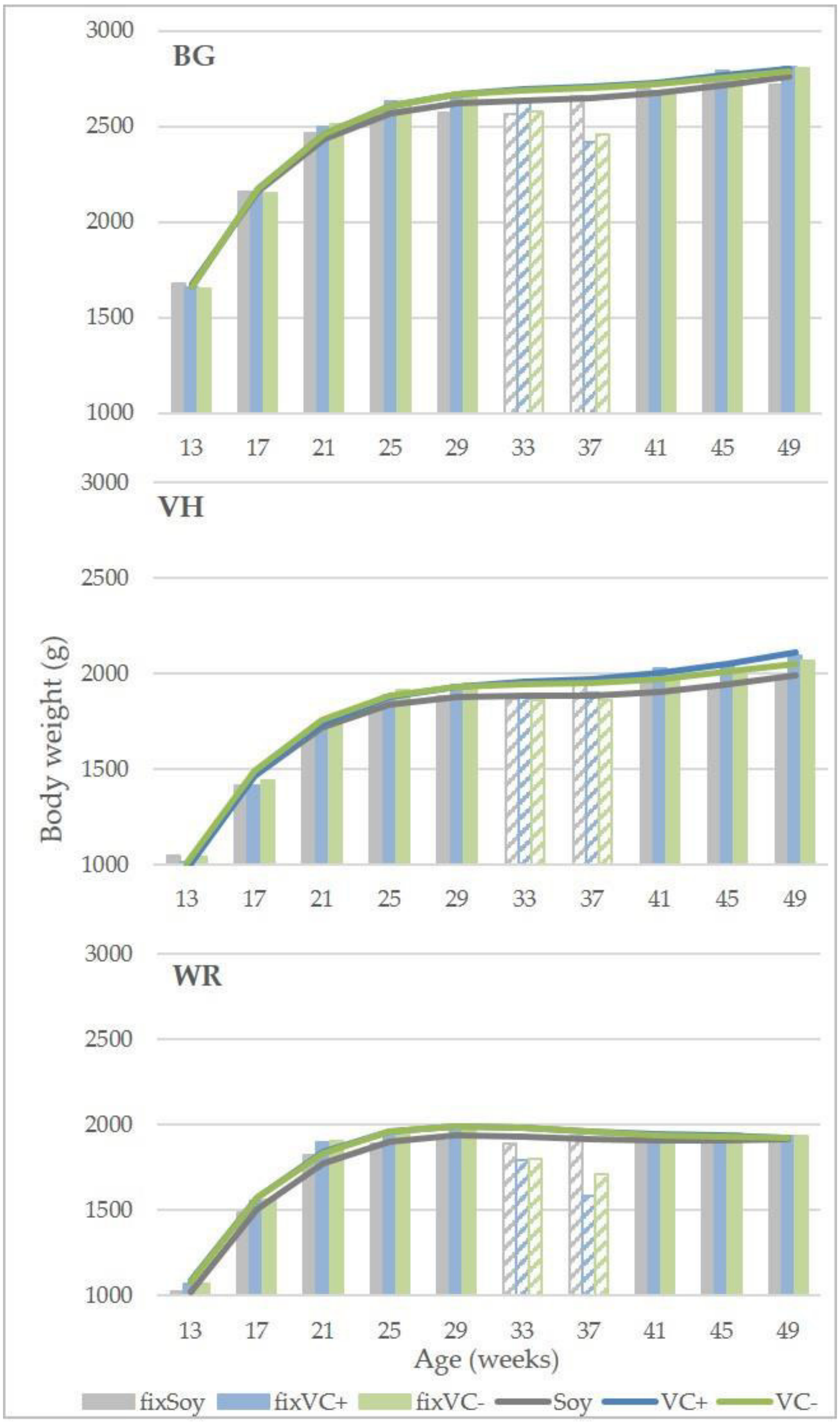

Figure S2. Modeling body weight of hens. Bar diagrams represent the complete data set. Data of striped bars was excluded from the final model, because of massive discrepancy between expected and measured values during a mite infestation in the chicken population. The exclusion of data took place iterative. The final growth curves (lines) were calculated via polynomial regression
