# Supplementary material for: Egg Production and Bone Stability of Local Chicken Breeds and Their Crosses Fed with Faba Beans
Source: Animals (Basel). 2020 Aug 22;10(9):1480. doi: 10.3390/ani10091480 (PMC7552325; doi:10.3390/ani10091480)
Supplement: Supplementary file 1 [file animals-10-01480-s001.zip › Supplement_FigureS3.pdf]

Supplementary Material

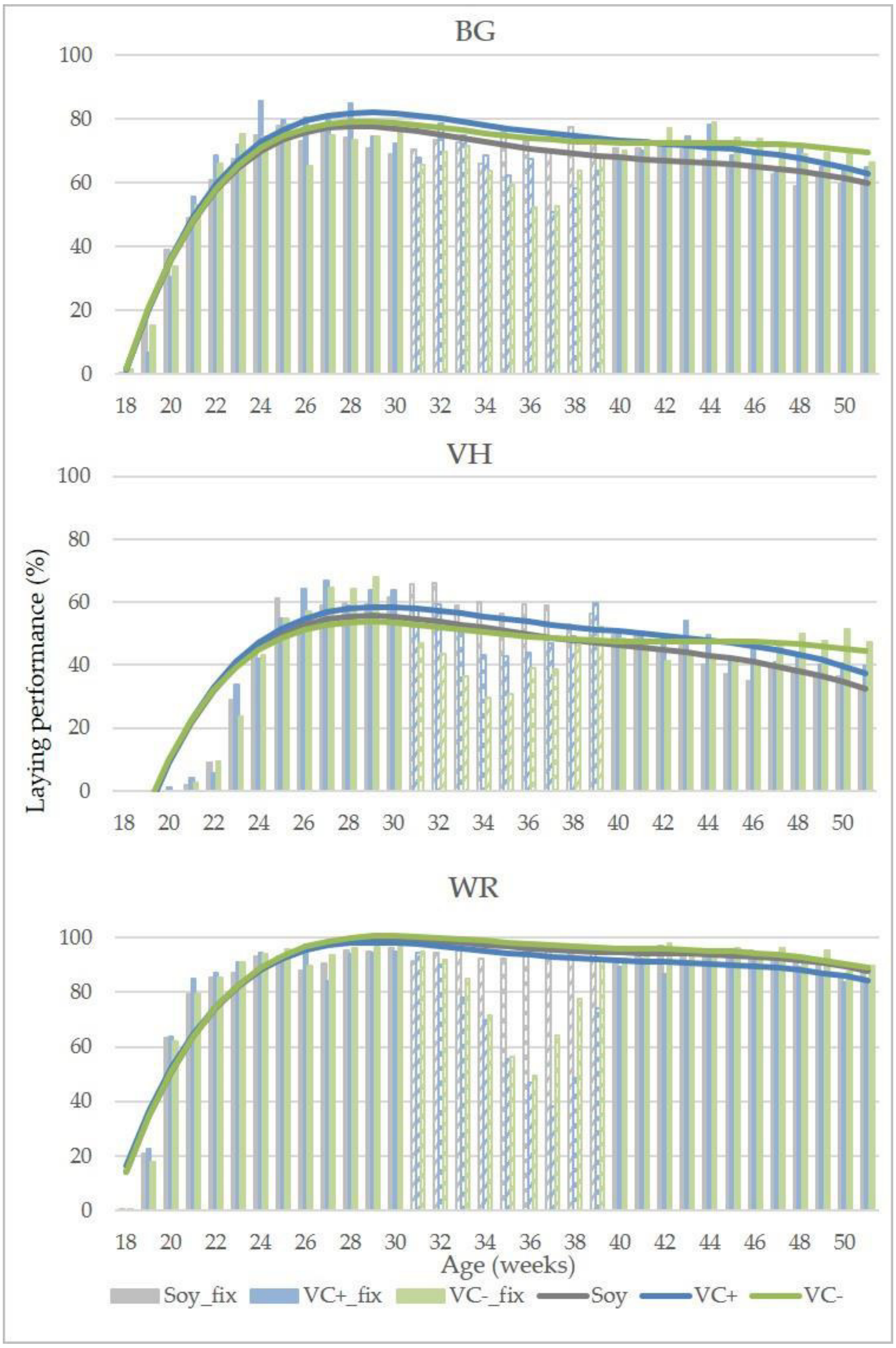

Figure S3. Modeling of laying performance. Bar diagrams represent the complete data set modeled with a linear mixed model. Data of striped bars was excluded from the final model, because of massive discrepancy between expected and measured values during a mite infestation in the chicken population. The exclusion of data took place iterative. The final curves (lines) were calculated via polynomial regression.
