# Supplementary material for: Egg Production and Bone Stability of Local Chicken Breeds and Their Crosses Fed with Faba Beans
Source: Animals (Basel). 2020 Aug 22;10(9):1480. doi: 10.3390/ani10091480 (PMC7552325; doi:10.3390/ani10091480)
Supplement: Supplementary file 1 [file animals-10-01480-s001.zip › Supplement_TableS1.pdf]

Supplementary Material

Table S1. Sample sizes of the analysis separated by genotype x diet combination.

	**BG**			**VH**			**WR**			**VBG**			**VWR**			**BWR**		
**Variable**	VC+	VC-	Soy	VC+	VC-	Soy	VC+	VC-	Soy	VC+	VC-	Soy	VC+	VC-	Soy	VC+	VC-	Soy
Bone breaking strength Tibiotarsus	39	35	32	39	39	37	39	38	40	43	44	44	44	44	43	44	44	44
Bone mineral density Tibiotarsus	39	35	32	39	39	37	39	38	40	44	44	44	44	44	43	44	44	44
Weight Tibiotarsus	39	35	32	39	39	37	39	38	40	44	44	44	44	44	43	44	44	44
Length Tibiotarsus	39	35	32	39	39	37	39	38	40	44	44	44	44	44	43	44	44	44
Thickness Tibiotarsus	39	35	32	39	39	37	39	38	40	44	44	44	44	44	43	44	44	44
Cortical area Tibiotarsus	39	35	32	39	39	37	39	38	40	44	44	44	44	44	43	44	44	44
Bone breaking strength Humerus	39	35	32	39	39	37	39	38	40	44	44	44	44	44	43	44	44	41
Bone mineral density Humerus	39	35	32	39	39	37	39	38	40	44	44	44	44	44	43	44	44	43
Weight Humerus	39	35	32	39	39	37	39	38	40	44	44	44	44	44	43	44	44	43
Length Humerus	39	35	32	39	39	37	39	38	40	44	44	44	44	44	43	44	44	43
Thickness Humerus	39	35	32	39	39	37	39	38	40	44	44	44	44	44	43	44	44	43
Bone mineral density Keel bone	39	35	32	39	39	37	39	38	40	44	44	44	44	43	43	44	44	43
Body weight	39	35	32	39	39	37	39	38	40	44	44	44	44	43	43	44	44	44

BG: Bresse Gauloise, VH: Vorwerkhuhn, WR: White Rock, VBG: VH male x BG female, VWR: VH male x WR female, BWR: BG male x WR female
